# Neuro-Inspired Signal Processing in Ferromagnetic Nanofibers

**DOI:** 10.3390/biomimetics6020032

**Published:** 2021-05-26

**Authors:** Tomasz Blachowicz, Jacek Grzybowski, Pawel Steblinski, Andrea Ehrmann

**Affiliations:** 1 Center for Science and Education—Institute of Physics, Silesian University of Technology, 44-100 Gliwice, Poland; 2 Faculty of Automatic Control, Electronics and Computer Science, Silesian University of Technology, 44-100 Gliwice, Poland; jacegrz498@student.polsl.pl; 3 Faculty of Electronics and Informatics, Koszalin University of Technology, 75-453 Koszalin, Poland; psteb@bobolin.com.pl; 4 Faculty of Engineering and Mathematics, Institute for Technical Energy Systems (ITES), Bielefeld University of Applied Sciences, 33619 Bielefeld, Germany; andrea.ehrmann@fh-bielefeld.de

**Keywords:** neuromorphic computing, nanofibers, bending radius, data processing, spikes, neuron excitation

## Abstract

Computers nowadays have different components for data storage and data processing, making data transfer between these units a bottleneck for computing speed. Therefore, so-called cognitive (or neuromorphic) computing approaches try combining both these tasks, as is done in the human brain, to make computing faster and less energy-consuming. One possible method to prepare new hardware solutions for neuromorphic computing is given by nanofiber networks as they can be prepared by diverse methods, from lithography to electrospinning. Here, we show results of micromagnetic simulations of three coupled semicircle fibers in which domain walls are excited by rotating magnetic fields (inputs), leading to different output signals that can be used for stochastic data processing, mimicking biological synaptic activity and thus being suitable as artificial synapses in artificial neural networks.

## 1. Introduction

Neuro-inspired signal processing presently relates to the most intensively studied topics of technical sciences in a wide range of completely different realizations. It can be implemented from an electronic perspective, using, for example, field-programmable gate array (FPGA) circuits [1,2,3,4] or employing the recent achievements of spintronics [5,6,7], phase change materials [8,9] or other components.

Different computational schemes have been developed, such as artificial neural networks (ANNs), support vector machines or reservoir computing as a special type of recurrent ANN [10,11,12]. Simulating the human brain with recurrent neural networks necessitates calibration of weights and connections of the nodes in the network; this time-consuming task can be avoided by using a reservoir in which weights between input values and reservoir neurons are either set arbitrarily or optimized [13,14], while the weights between reservoir neurons and output layer are linearly trained, thus speeding up the training process [15,16]. For all these approaches, it is necessary to produce artificial neurons and synapses that are able to transfer and modulate data. Often, such artificial neurons and synapses are produced by electronic devices with a variable resistance which represents the synaptic weight [17,18,19].

Several approaches to neuro-inspired signal processing are based on magnetic materials. There is a rich tradition of such research, firstly introduced by works of Allwood, Cowburn et al. [20,21,22] and more recently by Grollier et al. [23,24], to name just a few. Generally, signal processing necessitates a deterministic or stochastic correlation between input and output values. This means that not only input and output need to be defined, but also data transport and processing between them. In several approaches, nanofibers or nanofiber networks are used for these tasks, in which data can be transported and manipulated in the form of domain walls [25,26,27]. The static and dynamic magnetic properties of such nanofibers depend strongly on their geometry, material and orientation with respect to the external magnetic field [28,29,30,31].

From the perspective of neuroscience and neuronal spikes, the effect of neuron excitation if a given threshold level is exceeded is fundamental [32,33,34]. In the language of bioelectronics, this can be interpreted in the following way: If the excitations from several inputs overlap constructively, a defined energy barrier is overcome, and the output action potential is different from zero. Hence, since biological signals have different amplitudes, it can be imagined that the implementation of a small network of ferromagnetic fibers might be a good choice to use magnetization dynamics to provide a wide enough range of vector values, mimicking neuronal and synaptic activity.

Here we investigate three-semicircle systems with two inputs and three outputs with dynamic micromagnetic simulations. Our results show that, depending on the chosen threshold values, different data processing operations can be defined in this simple system. It must be mentioned that the nanofiber network presented here forms a single artificial neuronal cell body, receiving signals and performing data processing, which can be used as a part of a forward-propagating neural network [35,36,37]. It does not form a full artificial neural network with axons and synapses, which has to be implemented after establishing the functionality of these devices. Generally, the functionalities of the elements found in the human brain are not 100% reflected by such a neuromorphic approach.

## 2. Materials and Methods

**Figure 1:**
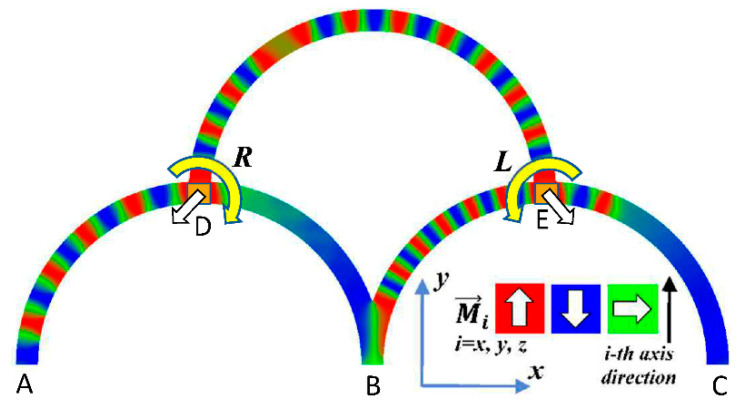
Simulated geometry, consisting of three magnetic half-circles with outputs (**A**–**C**) and inputs (**D**,**E**). The orientation of the magnetization is depicted by the color-code given in the inset: red = up, blue = down, green = horizontal.

Micromagnetic simulations were carried out using the micromagnetic solver Magpar, which dynamically solves the Landau–Lifshitz–Gilbert equation of motion [38]. The simulation parameters were chosen as follows: saturation magnetization $J_{S}=1.005\;T$, damping constant α=0.02, exchange constant $A=1.3\cdot 10^{-11}\;J/T$, and anisotropy constant equal zero since the material permalloy (Py) was chosen. The total length of each single fiber is 1570 nm with a bending radius of 500 nm, the cross-section 10 nm × 60 nm, meshed with an average size of 3.3 nm. The externally applied field is 1 T at a frequency of 500 MHz, applied at local input areas of 60 nm × 60 nm × 10 nm. The simulated geometry is depicted in Figure 1. This combination of material and dimensions was investigated before and found to be suitable to enable domain wall nucleation and propagation by a local rotating magnetic field of the frequency chosen here [39]. Such nanostructures can be produced by e-beam lithography or similar methods from different materials, typically on Si wafers or other non-magnetic flat surfaces, but also in a free-standing form [40,41,42].

Micromagnetic simulations are performed with four input combinations, defined by in-plane rotating fields: LL, LR, RL, and RR, where L = counterclockwise and R = clockwise. These inputs are used to feed information into the system. The resulting output signals are derived at the positions A, B, and C, meaning that we obtain magnetization vectors. Each vector component can have values from −1 to +1. After that, for a given component (separately for $M_{x},\;M_{y},\;M_{z}$) the weighted sum is calculated. This step is not yet implemented physically; possible realizations are connections with different lengths, diameters or in general different probabilities for data transport in a defined time.

It should be mentioned that while this is a proof-of-concept, building up the system in reality is technologically possible. The dimensions chosen here are relatively large and well suited for lithographic production. Applying localized varying magnetic fields at regions of some ten to a few hundred nanometers diameter is usually performed by microwave technique, as shown in diverse papers [43,44,45,46].

## 3. Results and Discussion

As an example, the calculation

(1) $$M_{x}^{\left(tot\right)}=w_{A}M_{x}^{\left(A\right)}+w_{B}M_{x}^{\left(B\right)}+w_{C}M_{x}^{\left(C\right)},$$

imitates similar combinations found in neural networks, with $w_{i}$ defining the weights of the respective outputs. The total signal $M_{x}^{\left(tot\right)}$ is then normalized by its maximum value that occurs during simulations, related usually to 150 ns or 200 ns. In this way, the $M_{x}^{\left(tot-norm\right)}$ component values fall in the range of <−1;+1>. Next, for the assumed threshold value $M_{th}$, a digitization operation is performed; i.e. the transformation from $M_{x}^{\left(tot-norm\right)}$ into $M_{x}^{\left(tot-dig\right)}$, namely

(2) $$M_{x}^{\left(tot-dig\right)}=\{\begin{array}{ccc} 1 & if & M_{x}^{\left(tot-dig\right)}\geq M_{th}\\ 0 & if & M_{x}^{\left(tot-dig\right)}<M_{th} \end{array}.$$

**Figure 2:**
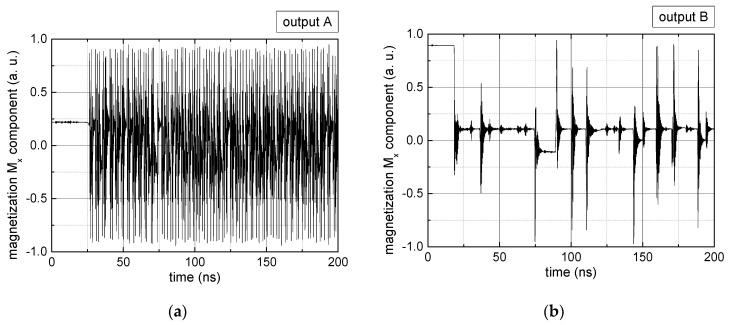

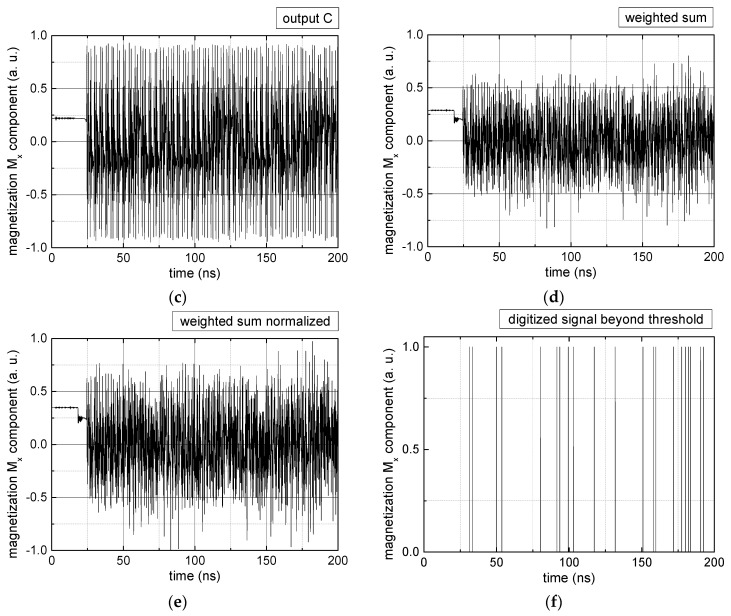
Data preparation steps: (**a**) time-resolved output of positions A, (**b**) B and (**c**) C; (**d**) the weighted sum over these outputs, as calculated in Equation (1); (**e**) the normalized weighted sum; and (**f**) the digitized signal which is higher than a defined threshold.

Since $M_{x}^{\left(tot-norm\right)}$ ∈ <−1;+1>, we tested $M_{th}\in \;<-1;+1>$ with a resolution of 0.1. The steps of data preparation are shown in Figure 2 for $w_{A}=w_{C}=0.45,\;w_{B}=0.1,$ $M_{th}=0.7$, and RL combination of rotating fields.

Firstly, Figure 2a–c shows the single outputs. It is visible here that outputs A and C show very fast oscillations between maximum and minimum x components, i.e., fast-moving and oscillating domain walls (cf. domain walls near output A in Figure 1). Output B behaves differently, as also visible in Figure 1. Here, the interaction between the left and the right semi-circle results in a more stable situation, with some “spikes” visible when domain walls move through this output. It should be mentioned that these spikes are not directly suitable to be used as logic results, as in spin-torque oscillators used to prepare neural circuits [47]. Instead, weighted sums of the three outputs are applied here to allow for differentiation between the input combinations LL, LR, RL and RR.

In all three outputs, it is visible that the signal starts only at approx. 20–25 ns. This time gap between the onset of the rotating input fields and the onset of receiving an output value is correlated with the velocity of the domain walls moving through the nanostrips (cf. Figure 1). After this time gap, the initial starting configuration is overwritten with the introduced signals.

Due to the spatial symmetry of our fiber-based neuron, for all simulated cases we assume $w_{A}=w_{c}\neq w_{B}$, while $w_{A}+w_{B}+w_{C}=1$. As an example of calculation results, we present in Table 1 the case RL along with $w_{A}=w_{c}=0.45,\;w_{B}=0.1;\;w_{A}=w_{c}=0.40,\;w_{B}=0.2$; or $w_{A}=w_{c}=0.35,\;w_{B}=0.3$, for several representative threshold values of $M_{th}$.

Here, the influence of the threshold values is obvious. Smaller values of $M_{th}\;$are easier overcome by $M_{x}^{\left(tot-dig\right)}$, so that smaller threshold values will lead to more positions being 1 than 0 and vice versa. In this way, it is possible to define the output with the desired probability.

This, on the other hand, is the basis for the common process of adding up signals. For this, it is necessary that not only “learning” is realized, but also “forgetting”; i.e., if a certain stimulus value (here named in this way to avoid confusion with the threshold values defined before) is not reached after a certain time, the sum of the signals is set back to its original value (here zero) and summing up starts again [48]. This process can be realized, e.g., by a mono-domain magnetic tunnel junction in which the input stimuli frequency must be high enough to allow for crossing the energy barrier that separates two stable states [49]. Quite similarly, here it is possible to define a certain time (i.e., number of simulation steps) after which a stimulus value must be crossed; else the state reached is “forgotten” and the process starts again from zero.

**Figure 3:**
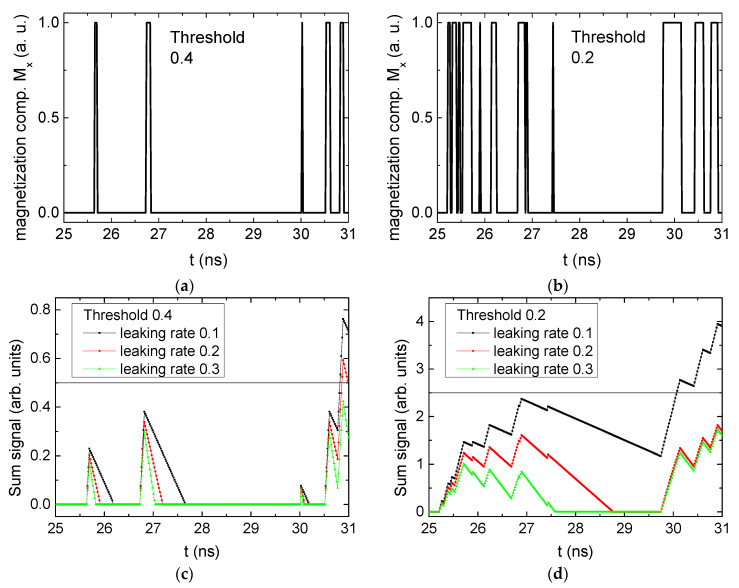
Magnetization components calculated for $w_{A}=w_{c}=0.35,\;w_{B}=0.3$ and threshold values of (**a**) *M_T_* = 0.4; (**b**) *M_T_* = 0.2; summing up signals for different leaking rates, calculated for the same weights and threshold values of (**c**) *M_T_* = 0.4; (**d**) *M_T_* = 0.2. Leaking rates are defined as “forgetting” rates; i.e., after 5 steps with a leaking rate of 0.2, a single “learning” step is “forgotten” again.

Figure 3 depicts the corresponding summing mechanism of two examples using the weights $w_{A}=w_{c}=0.35,\;w_{B}=0.3$ and thresholds of 0.4 or 0.2, respectively. The first 25 ns in which the input signals do not fully influence the output signals are neglected. While Figure 3a,b show the original signals derived for these thresholds, Figure 3c,d show the corresponding “learning and forgetting” simulation. Here, each “1” in Figure 3a,b adds a defined value in Figure 3c,d (“learning”), while for each “0” in Figure 3a,b, a value > 0 in Figure 3c,d is reduced by a defined value (“forgetting”). The leaking rate, defining the “forgetting”, is set to values from 0.1 to 0.3. A value of 0.1, e.g., means that one “learning” step” is “forgotten” after 10 “forgetting” steps, etc. 

Comparing these exemplary threshold values (cf. Table 1), it becomes clear that they should be correlated with different stimulus values, here chosen as 0.5 (Figure 3c) or 2.5 (Figure 3d), respectively, as marked by the horizontal lines. Comparing “learning” (i.e., ranges of increasing values) and “forgetting” (i.e., ranges of decreasing values), it can be noted that the first shows a certain stochastic behavior, as mentioned before, while “forgetting” is here realized by a simple linear function, as explained in the caption of Figure 3. This can be modified in a next step to mimic the human brain more adequately; however, this was not within the scope of this project.

While until now we concentrated on the input case RL to describe the system and its basic functionality, a discussion of the influence of the input on the weighted output is still necessary. Table 2, Table 3 and Table 4 depict the values of the digital signals, averaged over the time in which a signal can be measured at the outputs (i.e., after 25 ns, cf. Figure 2), derived for the different input cases LL, LR, RL and RR in case of the aforementioned combinations of weights and threshold values. It must be mentioned that these weights are just a few from a broad range of possible combinations. The column RL corresponds to the values depicted in Figure 2; Table 2, Table 3 and Table 4 correspond to the weight combinations in columns 2–4 of Table 1.

In particular, a threshold value of 0 (marked in grey) seems to be suitable to differentiate between different input combinations. It must be mentioned that the differences in the averaged values found for this threshold value (and others) strongly depend on the chosen weights. While for a combination of $w_{A}=w_{c}=0.45,\;w_{B}=0.1$, i.e., the smallest value of $w_{B}$ chosen here, LL and RR give quite similar results for *M_th_* = 0, LR and RL differ clearly from each other and from the symmetric cases (Table 2). This is different for the cases in which larger values for $w_{B}$ were chosen (Table 3 and Table 4), where LL and RR give quite different results, while LL and RL (Table 3) or LL and LR (Table 4) give nearly identical averaged values.

As this short overview shows, the system suggested here cannot be used to build up a classical binary logic, as it is available in a transistor, etc. Instead, a more complicated logic is enabled with a broad range of possible correlations between inputs and outputs, defined by the combinations of weights of the single outputs. Similar to artificial neural networks, setting these weights will define the results of the performed logic operations, i.e., the correlation between inputs and output. In a full neural network, the averaged values can also be used to define new weights in the next layer.

As these examples show, domain wall motion in small nanowire networks can serve to simulate neuronal behavior, including “learning” and “forgetting”.

## 4. Conclusions

In a recent study, neurons were defined as three coupled semicircle fibers in which domain walls are excited by rotating magnetic fields. These inputs, defined by pairs or rotational orientations (clockwise/counterclockwise), result in different outputs, which were investigated in terms of “learning” and “forgetting”. Depending on the number of added signals per time, defining “learning”, and the leaking rate, defining “forgetting”, these artificial neurons are found to reach a defined stimulus value with a certain probability. Such simple systems, which can be prepared by lithographic processes, can thus be used as parts of neuromorphic hardware.

## Figures and Tables

**Table 1:** Digital signals, derived for the case RL and different combinations of weights and threshold values, as explained in the text. The x-axes differ to make the signals better visible.

$M_{th}$	$w_{A}=w_{c}=0.45,\;w_{B}=0.1$	$w_{A}=w_{c}=0.40,\;w_{B}=0.2$	$w_{A}=w_{c}=0.35,\;w_{B}=0.3$
0.8	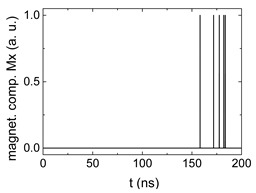	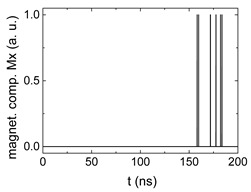	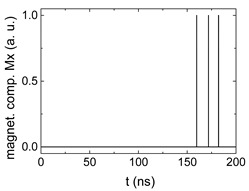
0.4	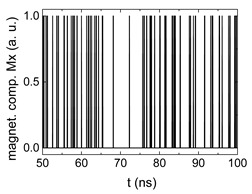	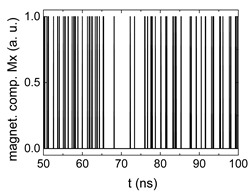	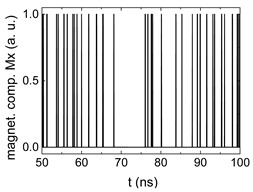
0	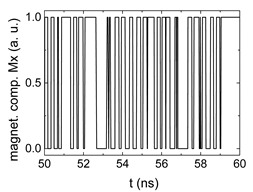	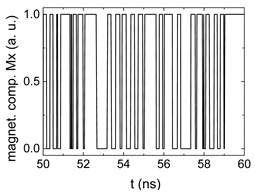	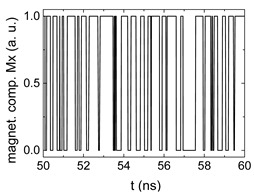
−0.4	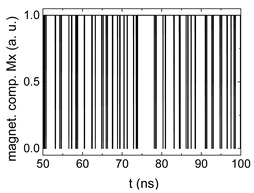	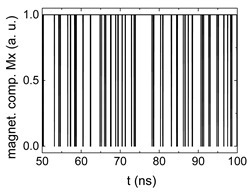	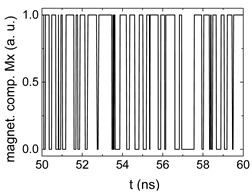
−0.8	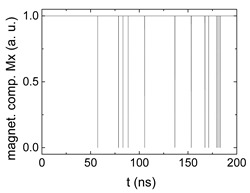	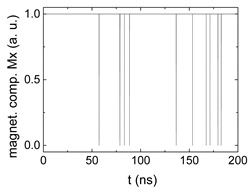	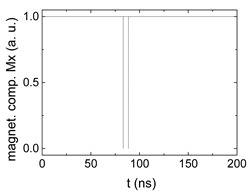

**Table 2:** Averaged values of digital signals for the weights $w_{A}=w_{c}=0.45,\;w_{B}=0.1$.

*M_th_*	LL	LR	RL	RR
+0.8	0.0032	0.0022	0.0015	0.0008
+0.4	0.0487	0.0591	0.0681	0.0809
0.0	0.3010	0.4839	0.5593	0.3169
−0.4	0.9867	0.9330	0.9452	0.9839
−0.8	0.9997	0.9985	0.9981	1.0000

**Table 3:** Averaged values of digital signals for the weights $w_{A}=w_{c}=0.40,\;w_{B}=0.2$.

*M_th_*	LL	LR	RL	RR
+0.8	0.0010	0.0022	0.0021	0.0019
+0.4	0.1431	0.0607	0.1426	0.1976
0.0	0.5754	0.4820	0.5754	0.8356
−0.4	0.9750	0.9286	0.9495	0.9982
−0.8	0.9993	0.9980	0.9987	1.0000

**Table 4:** Averaged values of digital signals for the weights $w_{A}=w_{c}=0.35,\;w_{B}=0.3$.

*M_th_*	LL	LR	RL	RR
+0.8	0.0062	0.0018	0.0013	0.0032
+0.4	0.1630	0.0526	0.1226	0.3047
0.0	0.4832	0.4811	0.5940	0.9532
−0.4	0.9999	0.9365	0.9704	1.0000
−0.8	1.0000	0.9989	0.9999	1.0000

## Data Availability

The data presented in this study are available on request from the corresponding author.
